# Expression and Purification of the Pain Receptor TRPV1 for Spectroscopic Analysis

**DOI:** 10.1038/s41598-017-10426-7

**Published:** 2017-08-29

**Authors:** Phanindra Velisetty, Richard A. Stein, Francisco J. Sierra-Valdez, Valeria Vásquez, Julio F. Cordero-Morales

**Affiliations:** 1 Department of Physiology, University of Tennessee Health Science Center, 71S. Manassas St., Memphis, TN 38163 USA; 2 Department of Molecular Physiology and Biophysics, Vanderbilt University Medical Center, Nashville, TN 37232 USA

## Abstract

The transient receptor potential vanilloid 1 (TRPV1) channel is an essential component of the cellular mechanism through which noxious stimuli evoke pain. Functional and structural characterizations of TRPV1 shed light on vanilloid activation, yet the mechanisms for temperature and proton gating remain largely unknown. Spectroscopic approaches are needed to understand the mechanisms by which TRPV1 translates diverse stimuli into channel opening. Here, we have engineered a minimal cysteine-less rat TRPV1 construct (eTRPV1) that can be stably purified and reconstituted for spectroscopic studies. Biophysical analyses of TRPV1 constructs reveal that the S5-pore helix loop influences protein stability and vanilloid and proton responses, but not thermal sensitivity. Cysteine mutants retain function and stability for double electron-electron resonance (DEER) and electron paramagnetic resonance (EPR) spectroscopies. DEER measurements in the closed state demonstrate that eTRPV1 reports distances in the extracellular vestibule, equivalent to those observed in the apo TRPV1 structure. EPR measurements show a distinct pattern of mobilities and spectral features, in detergent and liposomes, for residues at the pore domain that agree with their location in the TRPV1 structure. Our results set the stage for a systematic characterization of TRPV1 using spectroscopic approaches to reveal conformational changes compatible with thermal- and ligand-dependent gating.

## Introduction

The detection of painful stimuli involves ion channels that depolarize sensory neurons to elicit or intensify inflammatory pain^1^. One such channel is the transient receptor potential vanilloid 1 (TRPV1) expressed by primary afferent sensory neurons, where it contributes to the mechanisms underlying pain hypersensitivity^2^. TRPV1 is a polymodal ion channel activated by noxious heat (>42 °C), the pungent ingredient of “hot” chili peppers (capsaicin)^3^, animal toxins^4–6^, extracellular protons^7^, and modulated by proalgesic inflammatory agents (e.g., bradykinin^8^, bioactive lipids^9, 10^) produced in response to tissue injury. Because TRPV1 is an important therapeutic target^11^, it is crucial to determine the structural rearrangements that lead to channel opening by multiple stimuli.

Functional and structural studies have provided insights into TRPV1 activation mechanisms by vanilloids and toxins^5, 12–18^. It is generally agreed that upon vanilloid binding, the interaction between capsaicin or resiniferatoxin [RTX] with the S4–S5 linker triggers rearrangements consistent with opening of the lower gate^14, 15^. Likewise, venom toxins (e.g., double knot toxin, DkTx) stabilize the open state by inserting hydrophobic residues into the membrane phospholipids and binding to the TRPV1 extracellular pore region^5, 16, 17^. Aside from vanilloids and toxins, acidification of the extracellular media can activate and/or potentiate TRPV1 responses to other stimuli^7, 13^. For instance, the heat response is potentiated by extracellular protons within the pH (6–7) range measured during tissue acidosis^7^. Three residues within the extracellular outer pore of TRPV1 (Glu600, Glu648, and Thr633) have been shown to be required for proton activation and modulation^7, 19^. Indeed, structures of TRPV1 in the apo and RTX/DkTx bound states suggest that protonation of Glu600 disrupts the interactions at the extracellular loops and promotes a conductive conformation of the selectivity filter^15^. Even though the proton binding site is known, the pH-dependent dynamic conformational rearrangements that couple the extracellular vestibule with the opening of the hydrophobic plug (lower gate) remain largely unknown. This mechanism can be further addressed by monitoring the allosteric conformational changes of TRPV1 using spectroscopic approaches.

Unlike with vanilloids and toxins, several TRPV1 regions and mechanisms have been proposed to account for thermal gating; and a consensus view has yet to emerge. For example, Voets *et al*. suggested that temperature sensitivity arises from the activation energy associated with voltage-dependent gating^20^. Chimeric and ortholog analyses support a role of the linker region (connecting the N terminus to the pore domain) and the N-terminal domain in thermosensitivity^21, 22^. On the other hand, involvement of the outer pore domain in heat sensitivity is supported by mutagenesis and modification of an external cation binding site^23, 24^. Others have shown that swapping C-terminal domains between TRPV1 and TRPM8 alters thermal sensing^25^. Another line of thought is that temperature sensitivity is driven by the solvation of hydrophobic residues during gating^26, 27^. Since heat sensitivity is an intrinsic property of TRPV1^28^, the stage is set for an in-depth analysis of its dynamic conformational changes during thermal gating using spectroscopic techniques such as metal ion fluorescence resonance energy transfer^29^ and electron paramagnetic resonance (EPR) spectroscopy.

EPR and double electron-electron resonance (DEER) spectroscopies have provided definitive mechanistic models for ion channels^30–35^, G protein-coupled receptors^36^, and transporters^37, 38^. These techniques have been mainly restricted to the examination of bacterial channels that contain few (or none) cysteine residues that are not critical for protein function; this requirement has largely hampered the study of mammalian ion channels using these approaches. Importantly, Salazar *et al*. engineered a functional full-length TRPV1 cysteine-less channel (18 cysteines mutated)^39^ that offers the possibility to express, purify, label, and measure dynamic conformational changes in TRPV1 during gating using EPR and DEER spectroscopies. Here, we have generated a functional minimal cysteine-less rat TRPV1 construct that can be stably purified, labeled, and reconstituted for spectroscopic analyses. Our work represents the initial step towards using EPR and DEER methods in TRP channels to measure mobilities and distance changes associated with polymodal gating. Functional and biochemical analyses of minimal cysteine-less rat TRPV1 constructs reveal that the S5-pore helix loop influences protein stability and vanilloid and proton responses, but not thermal sensitivity. We have determined that 35 single-cysteine mutants along the rat TRPV1 sequence are functional and could be used to monitor conformational changes during channel activation. TRPV1 EPR and DEER spectra in the closed state display a pattern of mobilities and distances that are consistent with their location in the apo cryo-electron microscopy structure (cryo-EM). Further insights into TRPV1 gating mechanisms could be achieved by monitoring dynamic conformational changes at different temperatures and pHs.

## Results

### Characterization of a functional minimal cysteine-less rat TRPV1 construct

**Figure 1:**
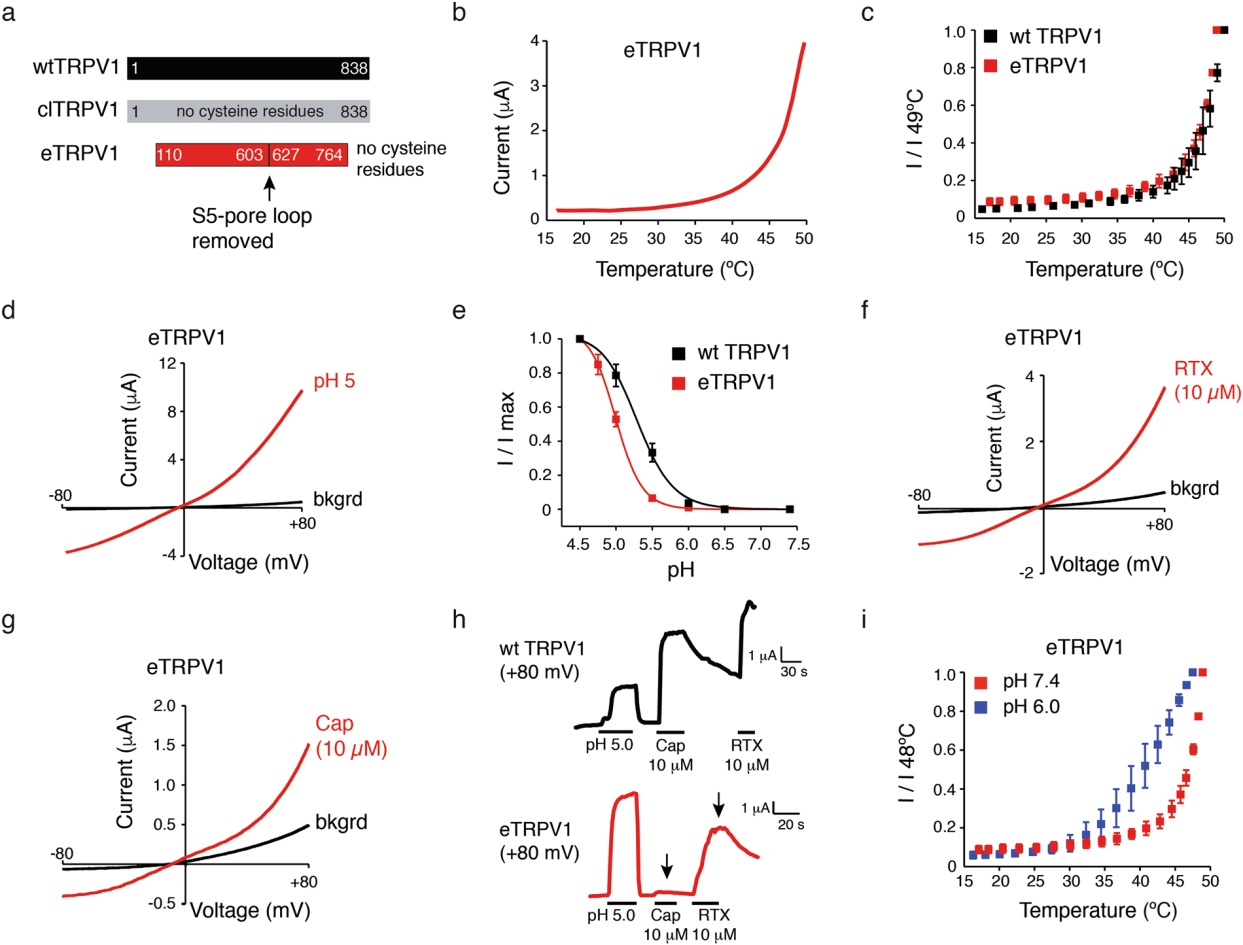
Characterization of a functional minimal rat cysteine-less TRPV1 construct (eTRPV1). (**a**) Schematic representation of eTRPV1 depicting the regions deleted (N- and C-termini and S5-pore helix loop) over the cysteine-less (cl) TRPV1 frame. (**b**) Heat-evoked currents of eTRPV1 extracted from voltage-clamp ramp protocols at +80 mV. (**c**) Temperature-response profile (+80 mV) of wt TRPV1 (black) and eTRPV1 (red); I/I 49 °C: Currents at each temperature/current at 49 °C. (**d**) Current-voltage relationship of eTRPV1 challenged with pH 5. (**e**) pH dose–response profiles (+80 mV) of wt TRPV1 and eTRPV1. I/I max: current at each pH/current at pH 4.5. n = 6. Squares are mean ± s.d. (**f**–**g**) Vanilloid-evoked currents of eTRPV1 challenged with 10 µM of resiniferatoxin (RTX) and capsaicin (Cap). (**h**) pH 5, Cap, and RTX-evoked currents of wt TRPV1 (top) and eTRPV1 (bottom) extracted from voltage-clamp ramp protocols at +80 mV. (**i**) Temperature-response profile (+80 mV) of eTRPV1 at pH 6.0 (blue) and 7.4 (red). I/I 48 °C: Current at each temperature/current at 48 °C. Background currents (bkgrd).

In the pursuit of spectroscopic approaches to TRPV1 gating, we engineered a minimal cysteine-less rat TRPV1 construct (referred to as eTRPV1 hereafter; “e” stands for EPR; Fig. 1a). A pivotal work reported^39^ the functional characterization of full-length cysteine-less rat (cl)TRPV1 (Fig. 1a). Unfortunately, in our hands this construct was not biochemically stable for spectroscopic studies (data not shown). Recently, a functional rat TRPV1 minimal construct was stably purified and used to obtain high-resolution structures^15, 16, 40^. We took advantage of these two templates to engineer eTRPV1, a construct that responds to both thermal and chemical stimuli, as determined by two-electrode volatge-clamp (TEVC) (Fig. 1). We found that eTRPV1 recapitulates the thermosensitivity observed in wt TRPV1 (Fig. 1b,c); thus, this construct can faithfully report the conformational changes that occur during thermal gating. Moreover, eTRPV1 elicited large outwardly rectifying currents when challenged with acidic pH (Fig. 1d), albeit with a small shift in the pH half-maximal response when compared with wild-type (wt) channels, EC_50_ = 5.0 ± 0.05 and 5.3 ± 0.004, respectively (mean ± CI, which indicates 95% confidence interval for the fit; Fig. 1e). Although eTRPV1 can be gated by RTX and capsaicin (Fig. 1f–g), we observed a significant decrease in the response to vanilloids when compared to wt TRPV1 (Fig. 1h, black arrow, and Supplementary S1a,b). These results were unexpected, as the vanilloid binding site^16^ at the pore domain (between S3, S4, and S5) was not modified in our construct. However, it is possible that cysteine substitutions, deletion of the S5-pore helix loop, or the combination of both could alter the allosteric coupling between the vanilloid binding site and the lower and upper gates. As reported for the chicken and zebrafish TRPV1 orthologs^12, 41^, as well as for mutations that affect capsaicin binding, our eTRPV1 construct is less sensitive to vanilloids but still maintains intact other gating modalities. As heat and protons are two of the main physiological stimuli, we conclude that eTRPV1 is a suitable tool for determining dynamic conformational changes using spectroscopic approaches.

The sensitization process is an intrinsic feature of TRPV1, since proalgesic agents such as pH 6 and fatty acids are able to lower the TRPV1 thermal activation transition in proteoliposomes^28^. Hence, we asked whether the eTRPV1 heat response could be sensitized by a subthreshold dose of proton concentration (pH 6, insufficient to activate the channel at room temperature; Fig. 1e). Indeed, heat-evoked currents exhibited a substantial shift in the thermal activation transition at pH 6.0 *vs*. pH 7.4 (35 °C and 42 °C, respectively; Fig. 1i), consistent with the proton potentiation of heat-activated currents observed in wt TRPV1-expressing oocytes and proteoliposomes^28, 42^. Thus, the eTRPV1 construct could be used to monitor conformational changes from closed to open transitions, as well as from closed to a putative sensitized state.

### Expression and purification of a stable minimal cysteine-less TRPV1 construct (eTRPV1)

**Figure 2:**
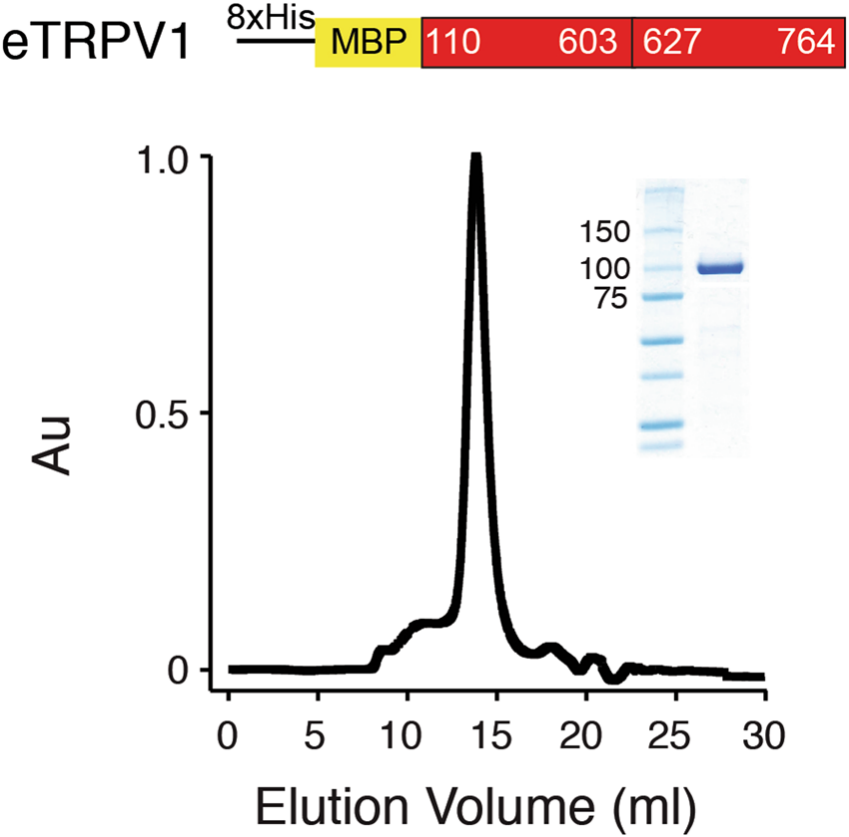
Purification of a stable minimal cysteine-less rat TRPV1 construct (eTRPV1). Top: schematic representation of the eTRPV1 construct used for insect cell expression. Bottom: size-exclusion chromatography profile of DDM-solubilized eTRPV1 protein after expression and purification from Sf9 cells. Inset: Stained protein on the SDS-PAGE gel corresponds to the size of the purified MBP-eTRPV1 monomer.

For expression and purification, we generated a baculovirus construct consisting of an 8× histidines-maltose binding protein (MBP) tag over the eTRPV1 sequence (Fig. 2; top). Next, we expressed eTRPV1 in insect cells (Sf9) and stably purified this construct to homogeneity. eTRPV1 migrates as a stable and pure monodisperse specie, as determined by size-exclusion chromatography (Fig. 2) and SDS-PAGE gel (Fig. 2; inset), respectively. Following this strategy, we obtained biochemical quantities of detergent-solubilized protein that can be labeled via cysteine covalent modification (e.g., fluorophores, spin-labeled [SL] methyl-methanethiolsulfonate) along the eTRPV1 sequence for spectroscopic analysis. Remarkably, our results are the first example of a functional TRP channel that can be stably purified in the complete absence of cysteine residues.

### The S5-pore helix loop influences channel vanilloid and proton responses, and protein stability

**Figure 3:**
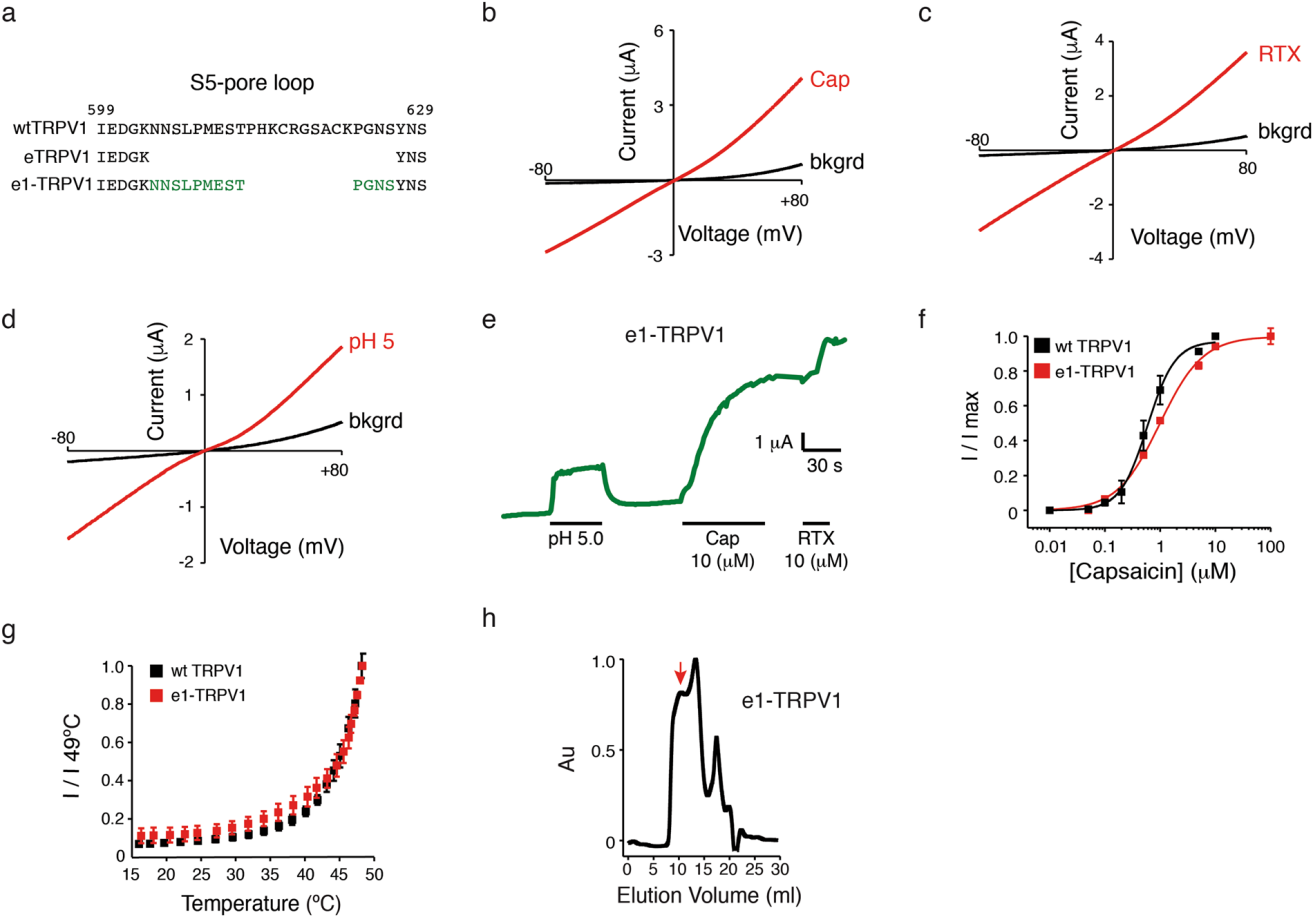
The S5-pore loop influences vanilloid sensitivity and protein stability. (**a**) Amino acid sequence highlighting the changes made in the S5-pore loop of eTRPV1 to generate e1-TRPV1 (green). (**b**–**d**) Representative current-voltage relationships of e1-TRPV1 challenged with capsaicin (Cap, 10 µM), resiniferatoxin (RTX, 10 µM), and pH 5. (**e**) pH 5-, Cap-, and RTX-evoked currents of e1-TRPV1, extracted from voltage-clamp ramp protocols at +80 mV. Note the increase in Cap and RTX responses as compared to pH. (**f**) Capsaicin dose-response profile of wt TRPV1 and e1-TRPV1 n = 5. Squares are mean ± s.d. (**g**) Heat-evoked currents of e1-TRPV1 extracted from voltage-clamp ramp protocols at +80 mV. (**h**) Size-exclusion chromatography profile of DDM-solubilized e1-TRPV1 protein after expression and purification from Sf9 cells. Note that large amounts of e1-TRPV1 elute in the void volume (red arrow).

To test whether the decreased sensitivity to vanilloids observed in eTRPV1 is due to the lack of the S5-pore helix loop, we added back 13 amino acid residues (from wt TRPV1) to the eTRPV1 construct and generated e1-TRPV1 (Fig. 3a, green residues). Unlike eTRPV1, e1-TRPV1 elicited robust outwardly rectifying currents when challenged with capsaicin, RTX, and pH (Fig. 3b-e). Indeed, the capsaicin dose-response curve for e1-TRPV1, EC_50_ = 0.94 µM ± 0.21, is similar to that of the wt TRPV1, EC_50_ = 0.59 ± 0.06 µM (mean ± CI, which indicates 95% confidence interval for the fit; Fig. 3f). As observed in eTRPV1, e1-TRPV1 recapitulates the thermosensitivity observed in wt channels (Fig. 3g). Even though e1-TRPV1 recovers vanilloid sensitivity, the purified protein elutes predominantly in the void volume, as determined by size-exclusion chromatography (Fig. 3h, red arrow); hence, this construct is not suitable for spectroscopic methods that require purified protein (e.g., EPR). Alternatively, e1-TRPV1 will be useful for detecting extracellular and intracellular conformational changes in heterologous expression systems using fluorescence-based techniques (e.g., patch-clamp and voltage-clamp fluorometry).

**Figure 4:**
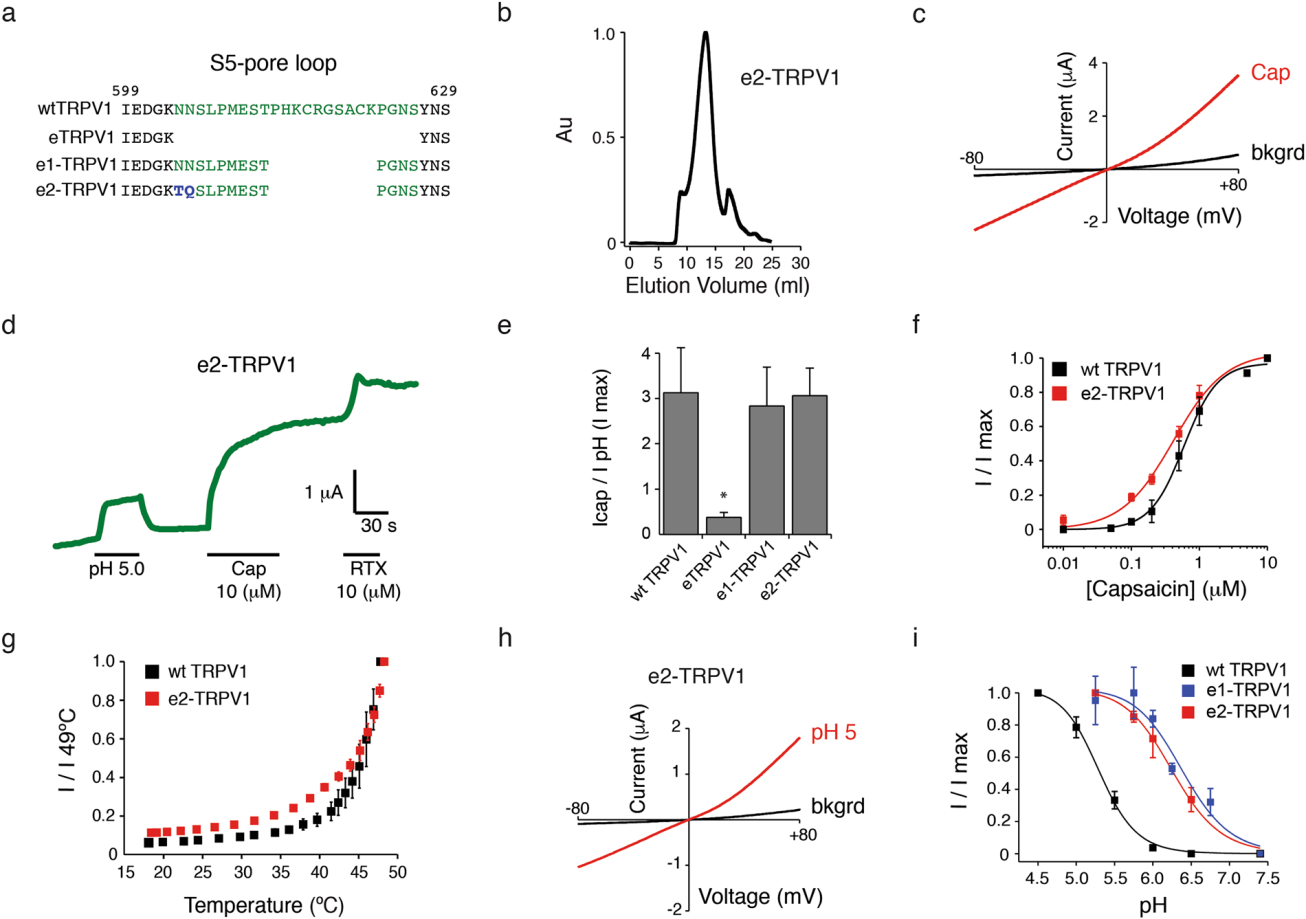
Asparagine residues at the S5-pore loop affect protein stability. (**a**) Amino acid sequence highlighting the changes made in the S5-pore loop of eTRPV1 to generate e2-TRPV1 (green and blue residues). e2-TRPV1 contains two mutations over the frame of the e1-TRPV1: (1) the glycosylation site was mutated to threonine (N604T) and (2) the neighboring residue mutated from asparagine to glutamine (N605Q). (**b**) Size-exclusion chromatography profile of DDM-solubilized e2-TRPV1 protein after expression and purification from Sf9 cells. Note that large amounts of e2-TRPV1 elute predominantly as a monodisperse peak (~13 ml). (**c**) Representative current-voltage relationships of e2-TRPV1 challenged with capsaicin (Cap, 10 µM). (**d**) pH 5-, Cap-, and RTX-evoked currents of e2-TRPV1, extracted from voltage-clamp ramp protocols at +80 mV. (**e**) Icap/IpH (I max): maximal currents of capsaicin/maximal current at pH 4.5 (+80 mV) of wt TRPV1, eTRPV1, e1-TRPV1, and e2-TRPV1. (**f**) Capsaicin dose-response profiles of wt TRPV1 and e2-TRPV1. n = 5. Squares are mean ± s.d. Charged residues at the S5-pore loop impact pH sensitivity of e1- and e2-TRPV1 constructs. (**g**) Heat-evoked currents of e2-TRPV1 extracted from voltage-clamp ramp protocols at +80 mV. (**h**) Representative current-voltage relationships of e2-TRPV1 challenged with pH 5. (**i**) pH dose–response profiles (+80 mV) of wt TRPV1, e1-TRPV1, and e2-TRPV1. I/I max: current at each pH/current at pH 5.5. n = 4–6. Squares are mean ± s.d. Kruskall-Wallis and Dunn’s multiple comparisons tests were used in (**e**). *p < 0.05.

TRPV1 has a glycosylation site (Asn604) at the S5-pore helix loop that could account for the instability of the e1-TRPV1 construct. Indeed, by mutating N604T and the neighboring residue N605Q (e2-TRPV1; Fig. 4a, blue residues), we found that e2-TRPV1 elutes as a monodisperse specie (Fig. 4b), recapitulating the biochemical properties observed in eTRPV1. Moreover, this biochemically stable construct also elicited robust currents when challenged with vanilloids (Fig. 4c–f and Supplementary S2) and had an EC_50_ for capsaicin comparable to that of wt TRPV1: EC_50_ = 0.42 ± 0.09 µM *vs*. 0.59 ± 0.06, respectively (mean ± CI, which indicates 95% confidence interval for the fit; Fig. 4f). Likewise, e2-TRPV1 displays thermosensitivity like that of wt channels (Fig. 4g). We next tested the pH sensitivities of e1- and e2-TRPV1 and found a shift in the pH half-maximal response when compared to wt channels: EC_50_ = 6.4 ± 0.23, 6.2 ± 0.08, and 5.3 ± 0.05, respectively (mean ± CI, which indicates 95% confidence interval for the fit; Fig. 4h–i), suggesting that less proton concentration is needed to open the channel. These results were unexpected because channel modifications did not include the pH regulatory sites (Glu600, Glu648, and Thr633)^7, 19, 43^. Additional studies will be needed to understand in detail the contribution of the loop in setting the pH threshold; nevertheless, we have generated a biochemically stable minimal cysteine-less TRPV1 construct (e2-TRPV1) that responds to all TRPV1 gating modalities. Our results so far suggest that the S5-pore helix loop influences channel vanilloid and proton responses and that the double asparagine in this region affects TRPV1 stability when purified from insect cells.

### Functional and biochemical analyses of eTRPV1 single-cysteine mutants

**Figure 5:**
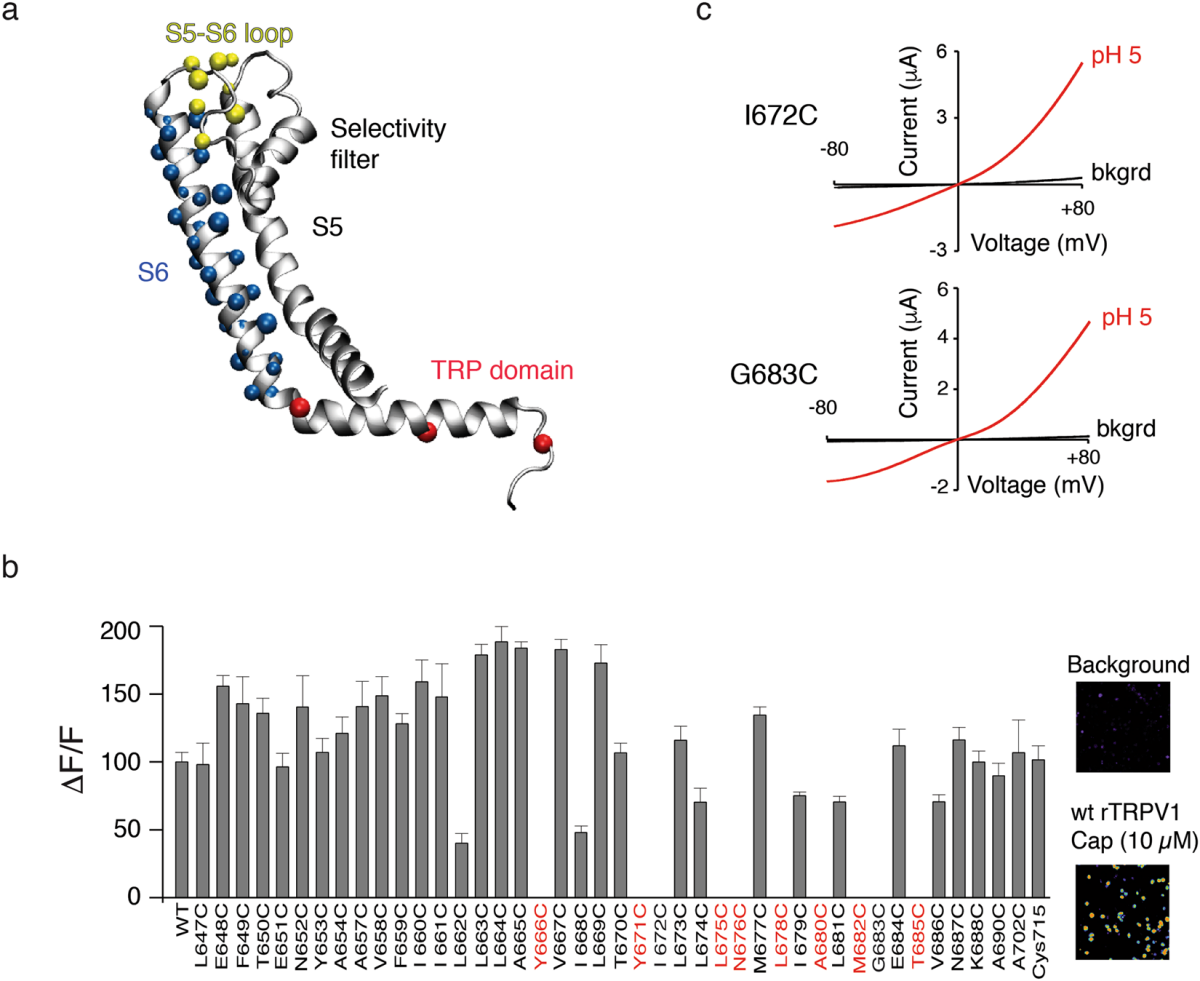
Functional analysis of eTRPV1 single-cysteine mutants evaluated in HEK293 cells by Ca^2+^ imaging and in *Xenopus *oocytes by TEVC. (**a**) One subunit (shown for clarity) of TRPV1 tetramer structure highlighting residues (colored spheres) substituted for single-cysteines. (**b**) HEK293 cell-expressing mutants (loaded with the Ca^2+^-sensitive Fluo-4-AM; 1 µM) were analyzed for capsaicin (10 μM)-evoked responses using fluorescence imaging (red denotes non-functional mutants in Ca^2+^ imaging). Right inset: representative capsaicin-evoked responses of control and wt TRPV1 transfected cells. (**c**) Current-voltage relationships of individual eTRPV1 single-cysteine mutants (I672C and G683C) challenged with pH 5 evaluated by TEVC. Background currents (bkgrd). Bars are mean ± s.d.

**Figure 6:**
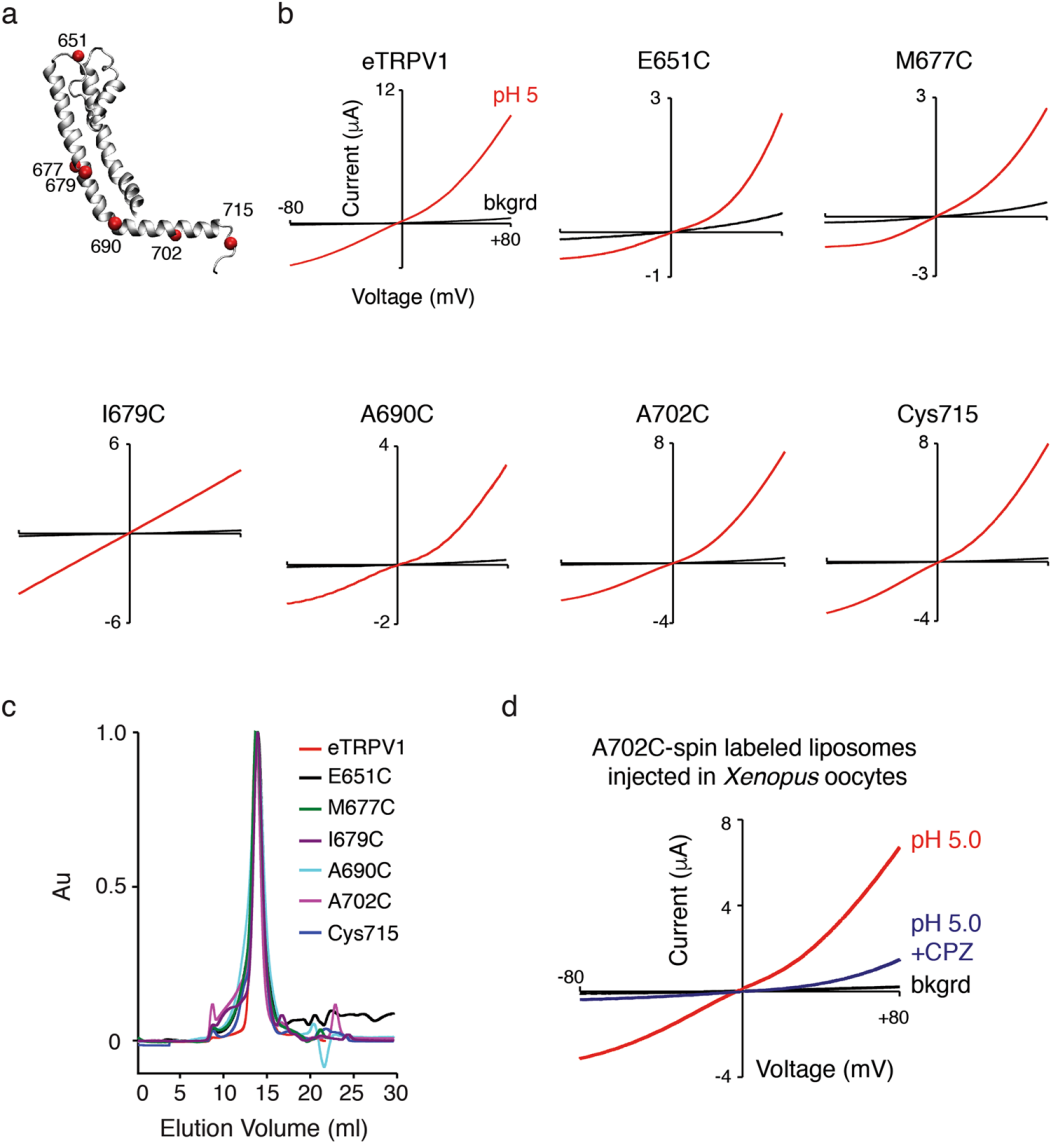
Purification of functional eTRPV1 single-cysteine mutants. (**a**) One subunit (shown for clarity) of TRPV1 tetramer structure highlighting single-cysteine residues (red spheres) introduced along the channel sequence and selected for purification. (**b**) Current-voltage relationships of eTRPV1 and individual eTRPV1 single-cysteine mutants (E651C, M677C, I679C, A690C, A702C, and the native Cys715) challenged with pH 5. (**c**) Size-exclusion chromatography profiles of DDM-solubilized eTRPV1 and eTRPV1 single-cysteine mutants after expression and purification from Sf9 cells (each color represents a different mutant). (**d**) Current-voltage relationships determined by TEVC from *Xenopus* oocytes microinjected with A702C-spin labeled-containing proteoliposomes challenged with pH 5 (red) and blocked by capsazepine (CPZ, blue: 40 µM). Background currents (bkgrd).

We have engineered and characterized three different TRPV1 constructs; however, eTRPV1 provides the most cost-effective protein yield for EPR experiments (>0.5 mg per liter of Sf9 cells). Because the EPR signal relies on the attachment of a paramagnetic spin-label to a cysteine residue, we generated 43 single-cysteine mutants along the eTRPV1 pore and TRP domain sequences (Fig. 5a) and tested their functionality with Ca^2+^ imaging in HEK293 cells (Fig. 5b). We found that 35 of the single-cysteine mutants were functional; hence, these positions can be spin-labeled to monitor conformational changes during channel activation. Since eTRPV1 display low response to vanilloids, we further tested the non-functional mutants by TEVC using pH 5. We found that I672C and G683C show robust proton-evoked currents, even though they appear insensitive to capsaicin (Fig. 5c). After the Ca^2+^ imaging experiments, we functionally and biochemically characterized representative positions along the TRPV1 pore and TRP domain (E651C, M677C, I679C, A690C, A702C, and Cys715; Fig. 6a). As observed in Fig. 6b,c, these mutants have large pH currents and migrate as stable monodisperse species, as analyzed by TEVC and size-exclusion chromatography, respectively. It is noteworthy that I679C lacks the outward rectification characteristic of TRPV1 (Fig. 6b), suggesting that disruption of the hydrophobic plug formed by the Ile679 at the lower gate^15, 40^ alters the conduction properties of the pore. To further assess the functionality of a purified and spin-labeled single-cysteine mutant, we reconstituted A702C-SL TRPV1 into asolectin liposomes and microinjected them into *Xenopus* oocytes. Note that pH 5 elicited robust outwardly rectifying currents (~7.5 µA; Fig. 6d) that were blocked by co-application of capsazepine (CPZ), a TRPV1 antagonist (Fig. 6d, blue trace). Acidic pH failed to evoke ionic currents in *Xenopus* oocytes microinjected with empty liposomes (data not shown). This result demonstrates that single-cysteine mutants can be stably purified while retaining their functionality after spin labeling.

### Structural dynamics of eTRPV1 spin-labeled single-cysteine mutants by DEER and EPR spectroscopies

**Figure 7:**
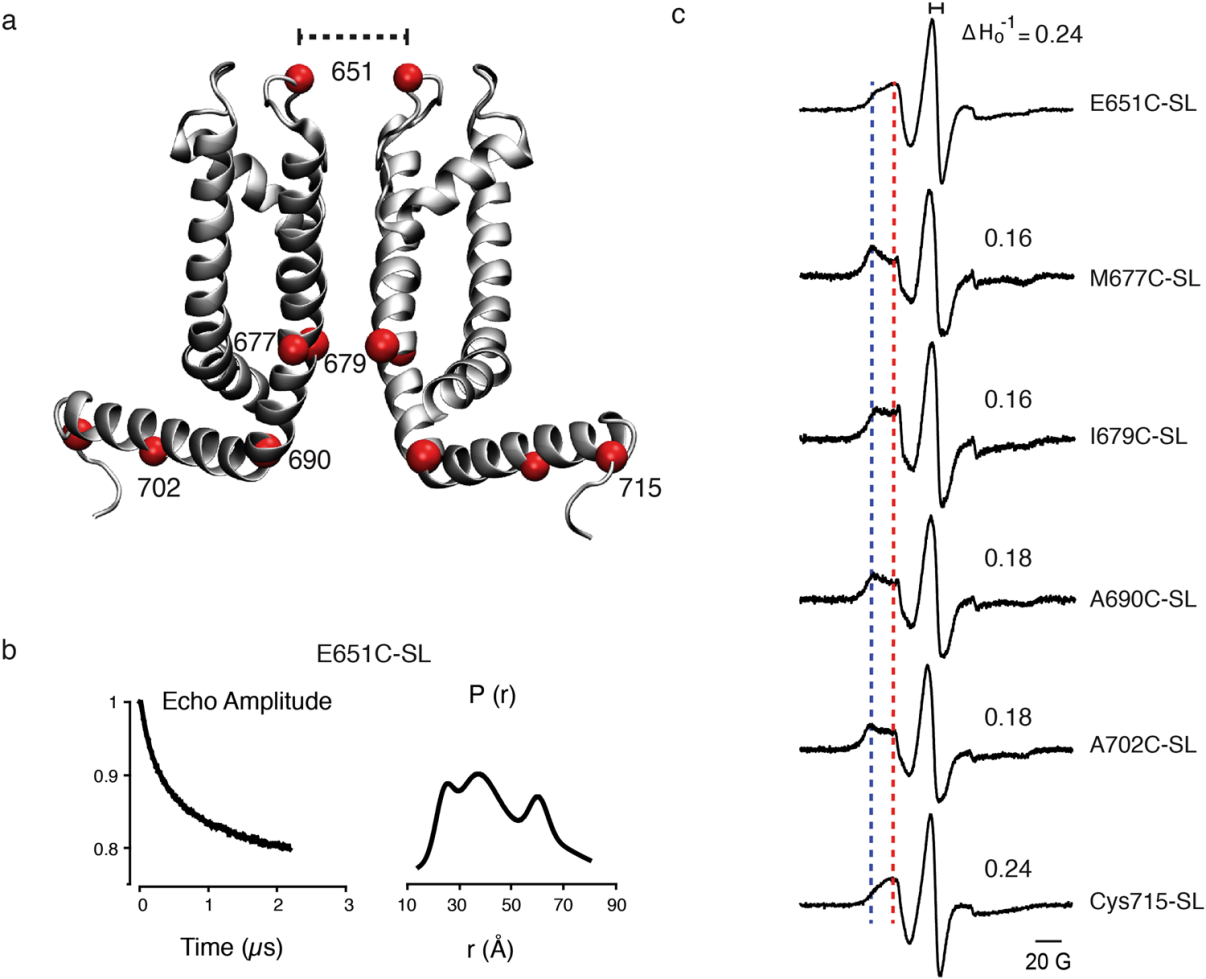
Distances and mobilities of spin-labeled eTRPV1 mutants in detergent. (**a**) Two subunits (shown for clarity) of TRPV1 tetramer structure highlighting the amino acid residues (red spheres) probed through site-directed spin labeling DEER and EPR spectroscopies. (**b**) DEER echo (left) and distance distribution (right) of spin-labeled mutant E651C. A sum of Gaussians was fit to the DEER data. (**c**) First-derivative of continuous-wave EPR spectra of spin-labeled cysteine mutants in DDM-solution. ΔH_o_^−1^ denotes the magnitude of the mobility parameter. The blue and red dotted lines highlight the immobile and mobile components of the spin-label spectra, respectively. EPR spectra were normalized to the total number of spin labels.

We sought to determine whether our eTRPV1 construct could report distances that correspond to the TRPV1 structure in the apo state using DEER spectroscopy. We centered our attention on the pore region, as this domain tends to be structurally conserved among the family of voltage-gated ion channels. To measure long-range intersubunit distances, we chose position Glu651, which, according to the cryo-EM structures^40^, is within the measurable distances for DEER (20–70 Å; Fig. 7a)^44^. Since there are four spin labels within the homotetramer, at least two distances were expected: one corresponding to the adjacent subunit and the other to the diagonal one. The histogram for Glu651 shows three peaks corresponding to 24, 36, and 58 Å (Fig. 7b). The two shorter distances are consistent with the apo TRPV1 structure (23 and 32 Å, for the Cβ-Cβ distances between adjacent and diagonal subunits, respectively)^40^, whereas the third 58 Å peak might correspond to protein aggregation. Given eTRPV1 quantity, quality, and pH sensitivity, this construct could be used to monitor distance changes occurring during proton gating.

The local environment surrounding a given residue modulates the dynamics of the spin-label; hence, probes exposed to the aqueous media display higher mobility values than those restricted to protein–protein and membrane–protein interactions. We used continuous-wave EPR to probe the dynamics of residues located at the pore domain (e.g., extracellular vestibule, hydrophobic plug, and TRP domain; Fig. 7a). The mobility parameter of the spin probe is calculated as the inverse of the central line width of the first derivative absorption spectra (ΔH_o_^−1^). This parameter is governed by the local environment of the spin label and the flexibility of the backbone to which it is attached^45^. As the motion of the spin label is reduced, the line width increases and ΔH_o_^−1^ decreases. ΔH_o_^−1^ in the closed state in solution (Fig. 7c) reveals a trend for the dynamics of the spin-labeled residues according to their location. Specifically, we measured higher mobility values for Glu651 (0.24) and Cys715 (0.24), residues exposed to the aqueous environment, than for Met677 (0.16), Ile679 (0.16), and Ala690C (0.18) residues in a proteinaceous environment at the intracellular gate. Position Ala702 (0.18) also exhibited restricted dynamics that might be due to its proximity to the linker domain, the pre-S1 and S1 helix. Our data are in agreement with mobilities reported for transmembrane helices of other ion channels^46–48^. A closer look at the first derivative absorption spectra components also revealed immobile spin populations (Met677, Ile679, A690C, and Ala702) at lower field strength and mobile populations (Glu651 and Cys715) at higher field strength (Fig. 7c, blue and red dotted lines, respectively). As anticipated, the mobilities observed for these positions are consistent with their location in the TRPV1 closed structure; hence, these parameters could potentially monitor dynamic changes (e.g., at the intracellular gate) during activation gating. For instance, we expect to observe an increase in mobilities at Met677 and Ile679 positions when eTRPV1 is challenged with acidic pH and/or heat.

**Figure 8:**
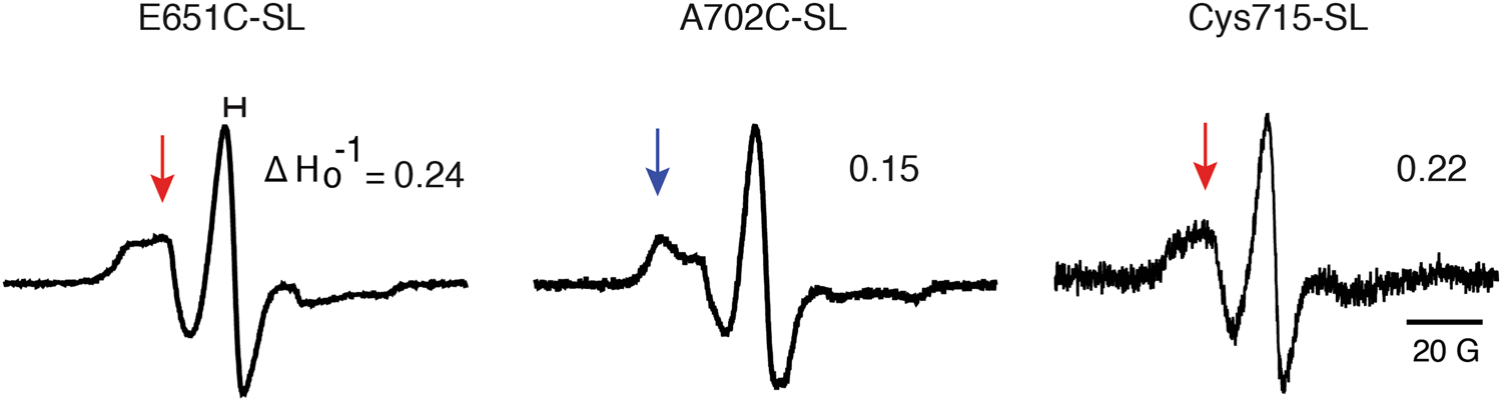
EPR spectra and mobilities of eTRPV1 spin-labeled cysteine mutants reconstituted in asolectin liposomes. Spectra were obtained at pH 7.4 (closed state). ΔH_o_^−1^ denotes the magnitude of the mobility parameter. The blue and red arrows highlight the immobile and mobile components of the spin-label spectra, respectively. EPR spectra were normalized to the total number of spin labels.

To assess the dynamic changes of TRPV1 in a membrane environment, we reconstituted spin-labeled mutants in asolectin liposomes. As expected for the closed state, the dynamic features of the spectra for eTRPV1-SL mutants did not change after reconstitution in liposomes, as residues exposed to the aqueous environment, Glu651 and Cys715, displayed large magnitudes for mobility (ΔH_o_^−1^ = 0.24 and 0.22, respectively) and a substantial contribution of mobile spin-label populations (Fig. 8, red arrows). Likewise, residue Ala702 conserves its sterically restricted features when reconstituted in liposomes (Fig. 8), as reflected by the extent of the spectrum broadening, mobility magnitude (ΔH_o_^−1^ = 0.15), and immobile spectra component (blue arrow). Taken together, our results demonstrate that TRPV1 can be purified, labeled, and reconstituted for spectroscopic analyses and that eTRPV1 is a properly folded protein as shown by distances and dynamic properties.

## Discussion

Functional and structural studies on TRPV1 have provided comprehensive information about its vanilloid^13–16^ and toxin^5, 6, 16, 17^ gating mechanisms. However, TRPV1 is a polymodal ion channel that responds to different physical and chemical stimuli, and likely via different pathways. Hence, TRPV1 is a prime candidate to use for an in-depth analysis of the dynamic conformational changes that occur during thermal- and proton-dependent gating using spectroscopic techniques. Dynamic information about TRPV1 embedded in a membrane environment is required to further understand its gating mechanism and lipid modulation. EPR and DEER spectroscopies have provided some of the most definitive mechanistic models for membranes proteins, for instance: the pH-dependent conformational changes in the bacterial K^+^ channel KcsA^35, 49^ and the pentameric ligand gated channel GLIC^47^, the conformational transitions of the voltage sensing domain in the archaeon K^+^ channel KvAP^30, 50^, and the conformational changes associated with ligand-dependent gating of the bacterial transporter LeuT^37^. Although these approaches have been mainly restricted to the examination of prokaryotic membrane proteins, current technologies for expression and purification of mammalian proteins allow the use of EPR and DEER to determine the resting state of a human proton channel in a lipid bilayer^51^, the gating mechanism of NMDA receptors^52^, and the conformational dynamics of a mammalian ABC transporter^38^.

To implement EPR and DEER, many difficulties must be overcome, including lack of protein function when removing all cysteine residues (especially important for mammalian proteins), low protein yield, protein instability during purification and after spin labeling, and protein aggregation in detergent or liposomes. Here, we have overcome these critical barriers and obtained DEER and EPR spectra information for a mammalian sensory receptor. We have engineered a minimal cysteine-less rat TRPV1 construct (eTRPV1) that displays wt thermal and proton sensitivities (but with reduced vanilloid responses) that can be stably purified, spin labeled, and reconstituted for spectroscopic approaches. DEER and EPR measurements show distance and mobility patterns consistent with their location and accessibilities according to the apo cryo-EM structure^40^. Additionally, we have generated two minimal cysteine-less TRPV1 constructs (e1- and e2-TRPV1) that respond to heat, protons, and vanilloids and that can be used for cell- or FRET-based applications, respectively. Biochemical and functional analyses of these minimal cysteine-less TRPV1 constructs revealed that the S5-pore loop influences protein stability, as well as vanilloid and proton responses. Why do TRPV1 constructs display decreased sensitivity to vanilloids (eTRPV1) and increased sensitivity to acidic pH (e1- and e2-TRPV1)? At this point, we can speculate that lacking the S5-pore loop (from residues 603 to 627) preceding the pore helix in eTRPV1 disrupts the coupling between the vanilloid binding pocket and the upper gate. Likewise, lacking charged residues at the S5-pore loop (i.e., His614, Lys615, Arg617, and Lys622) could alter the protonation state of Glu600 and increase pH sensitivity in e1- and e2-TRPV1 constructs; similar results have been observed when mutating Glu600 to lysine^7^.

TRP channels’ temperature sensitivity is one of the most fascinating and less understood gating mechanisms; hence, by means of spectroscopic approaches, it will be possible to determine how membrane proteins translate thermal energy into protein motion. Future experiments will be directed to determine TRPV1 conformational changes compatible with thermal- and ligand-dependent gating. Beyond TRPV1, our results will motivate future spectroscopic studies of other members of the TRP channel family (e.g., TRPV2 and TRPV6).

## Materials and Methods

### Molecular biology

The minimal rat TRPV1 construct (110–603 and 627–764)^40^ was built over the full-length rat TRPV1 cysteine-less channel^39^ using the PCR method with Phusion High-Fidelity DNA Polymerase (Thermo Fisher Scientific). Single**-**cysteine mutants were generated using the QuickChange® Lightning Site-Directed Mutagenesis Kit (Agilent Technologies). For oocyte expression, genes were subcloned into the combined mammalian/oocyte expression vector pMO (modified pcDNA3). For protein purification, genes were subcloned into the 8x-histidines-MBP-containing pFastBac vector (Thermo Fisher Scientific). All constructs were verified by DNA sequencing.

### Calcium imaging

WT TRPV1, eTRPV1, and eTRPV1 single-cysteine mutants were transiently expressed in HEK293 cells with Lipofectamine 2000® (Thermo Fisher Scientific). HEK293 cells were loaded with Fluo-4-AM (1 µM) and placed on coverslips coated with poly-L-lysine (Sigma-Aldrich). Images were acquired and analyzed with cellSens (Olympus Life Science).

### DNA linearization and mRNA preparation

DNA constructs (wt TRPV1, eTRPV1, e1TRPV1, and e2TRPV1) and single-cysteine mutants in pMO were linearized using *Pme*I. mRNAs were prepared from linearized DNAs using the Ambion mMESSAGE mMACHINE T7 kit (Thermo Fisher Scientific) and purified using the RNeasy mini kit (Qiagen) for oocytes injection.

### *Xenopus* oocyte electrophysiology

Oocytes from *Xenopus laevis* were purchased from Nasco and processed using 1 mg/ml collagenase (Worthington) in ND96 solution (96 mM NaCl, 2 mM KCl, 5 mM HEPES, 1 mM MgCl_2_; pH 7.4). Five ng of mRNA corresponding to wt TRPV1, eTRPV1, e1TRPV1, e2TRPV1, and eTRPV1 single-cysteine mutants were injected into oocytes using a nanoliter-injector system (Warner Instruments; Hamden, CT, USA). Oocytes were kept at 16 °C in ND96 supplemented with 2 mM CaCl_2_. TEVC measurements were performed 24–36 h after injection. Borosilicate glass pipettes (WPI) were filled with 3 M KCl and whole-cell currents were measured in a 120 mM NaCl, 2 mM KCl, 2 mM MgCl_2_, 1 mM EGTA, and 10 mM HEPES solution; pH 7.4. Data were acquired with an Axoclamp 900 A and analyzed with pCLAMP 10.4 software (Molecular Devices).

### Protein expression and purification

Recombinant baculoviruses of TRPV1 constructs and single-cysteine mutants were obtained following the Bac-to-Bac expression system’s protocol (Thermo Fisher Scientific). Protein expression and purification were performed following previous protocols^28^. Sf9 cells were infected with baculovirus, and cells were collected after 72 h by centrifugation. Cells were lysed with a hand-held glass homogenizer in 36.5 mM sucrose, 2 mM TCEP, and 50 mM Tris (pH 7.4) in the presence of protease inhibitors (1 mM PMSF, 3 µg/ml leupeptin, 3 µg/ml aprotinin, and 1 µg/ml pepstatin). Cell debris were removed by centrifugation at 8,000 *g* for 20 min, and membranes were collected by high-speed centrifugation at 100,000 *g* for 50 min. Membranes were solubilized in buffer 1 (150 mM NaCl, 2 mM TCEP, 10% glycerol, 50 mM HEPES; pH 7.4), protease inhibitors, and 20 mM DDM for 2 h at 4 °C. Insolubilized membranes were discarded by centrifugation at 100,000 *g* for 50 min. and supernatant was incubated with amylose resin for 2 h at 4 °C. Protein-bound amylose resin was washed with buffer 2 (150 mM NaCl, 10% glycerol, 20 mM HEPES, 0.5 mM DDM, 0.1 µg/ml asolectin, 0.5 mM TCEP; pH 7.4), followed by a second wash with buffer 2 but without TCEP (buffer 3). Protein was eluted with buffer 3 supplemented with 20 mM maltose.

### Site-directed spin-labeling

After elution, single-cysteine mutants were further concentrated up to 2 mg/ml for spin-labeling. Spin-labeling was performed by addition of a 10-fold molar excess (3 times, every 30 min) of (1-oxyl-2,2,5,5-tetramethylpyrrolidin-3-yl) methyl methanethiosulfonate (MTSSL) (Toronto Research Chemicals) from a 100-mM stock solution in DMSO. The reaction was kept in the dark at room temperature for 1 h 30 min, followed by overnight incubation at 4 °C. On the next day, proteins were run on a Superose 6 Increase 10/300 GL size exclusion chromatography column (GE, Healthcare Life Sciences) and equilibrated in buffer 4 (150 mM NaCl, 20 mM HEPES, 0.5 mM DDM; pH 7.4).

### Liposome reconstitution

Liposome reconstitution was performed following previous protocols^28^. Soybean polar lipid extract in chloroform (Avanti) was dried using a rotary evaporator for 1 h. Dried lipids were resuspended in buffer 5 (200 mM NaCl, 5 mM MOPS; pH 7.4) and sonicated to form a homogenous mixture. Liposomes were destabilized with 2 mM DDM for 30 min and mixed with purified minimal cysteine-less TRPV1 and/or respective single-cysteine mutants using a 1:5 (mass: mass) ratio. The mixture was adjusted close to the detergent critical micelle concentration (CMC) and incubated overnight at 4 °C with gentle agitation. The volume was doubled, and biobeads SM-2 (Bio-Rad) were added and replaced three times with gentle agitation during 1 h incubation to remove the detergent from the sample. Beads were removed by filtration, and the proteoliposomes were collected by ultracentrifugation at 100,000 *g* for 1 h. The pellet was resuspended in buffer 5 and used for TEVC or EPR measurements.

### Proteoliposomes-*Xenopus* oocyte electrophysiology

Fifty nl of different concentrations of A702C-spin label proteoliposomes were injected into *Xenopus* oocytes using the nanoliter injection system. TEVC measurements were performed 12 h post-injection. Data were acquired with an Axoclamp 900 A and analyzed with pCLAMP 10.4 software (Molecular Devices).

### DEER and EPR spectroscopies

For DEER measurements, spin-labeled eTRPV1 (at 50 μM) in buffer 4 were supplemented with 30% glycerol for cryo-protection. DEER measurements were performed on an Elexsys E580 (Bruker) pulsed EPR spectrometer operating at Q-band frequency (33.9 GHz) and equipped with a 10-W Amp-Q amplifier (Bruker) with the dead-time free four-pulse sequence at 83°(ref. 53). The pulse lengths were 10 ns (π/2) and 20 ns (π) for the probe pulses and 40 ns for the pump pulse. The frequency separation was 63 MHz. Primary DEER decays were analyzed using home-written software operating in the Matlab environment^54, 55^. Briefly, the software carries out global analysis of the DEER decays obtained under different conditions for the same spin-labeled position. The distance distribution is assumed to consist of a sum of Gaussians, the number of which is determined based on a statistical criterion. For continuous-wave EPR experiments, spin-labeled eTRPV1 samples were loaded in capillaries, and spectra were collected on a Bruker EMX spectrometer using a 10-mW microwave power level and a modulation amplitude of 1.6G. The EPR signals were normalized to the total number of spin labels, dividing the spectra by the peak-to-peak value of the double integral. Probe mobility was derived from the spectral line shape, determined as the inverse of the central line width of the first-derivative absorption spectra (ΔH_o_^−1^).

### Data availability

All data generated or analyzed during this study are included in this published article (and its supplementary information files).

## Electronic supplementary material

Supplementary Information
