# Supplementary material for: Expression and Purification of the Pain Receptor TRPV1 for Spectroscopic Analysis
Source: Sci Rep. 2017 Aug 29;7:9861. doi: 10.1038/s41598-017-10426-7 (PMC5575240; doi:10.1038/s41598-017-10426-7)
Supplement: Supplementary file 1 — Supplementary Information [file 41598_2017_10426_MOESM1_ESM.pdf]

Expression and Purification of the Pain Receptor TRPV1 for Spectroscopic Analysis

Phanindra Velisetty[1], Richard A. Stein[2], Francisco J. Sierra-Valdez[1], Valeria Vásquez[1], Julio F. Cordero-Morales[1]*

[1]Department of Physiology, University of Tennessee Health Science Center, 71 S. Manassas St., Memphis, TN 38163 USA.

[2]Department of Molecular Physiology and Biophysics, Vanderbilt University Medical Center, Nashville, TN 37232 USA.

*Correspondence: Julio F. Cordero-Morales (JFC-M)

University of Tennessee Health Science Center. 71 S. Manassas St. Memphis, TN 38163 USA

Telephone: 901-448-8206

Email: jcordero@uthsc.edu

Supplementary figures

Supplementary Figure S1

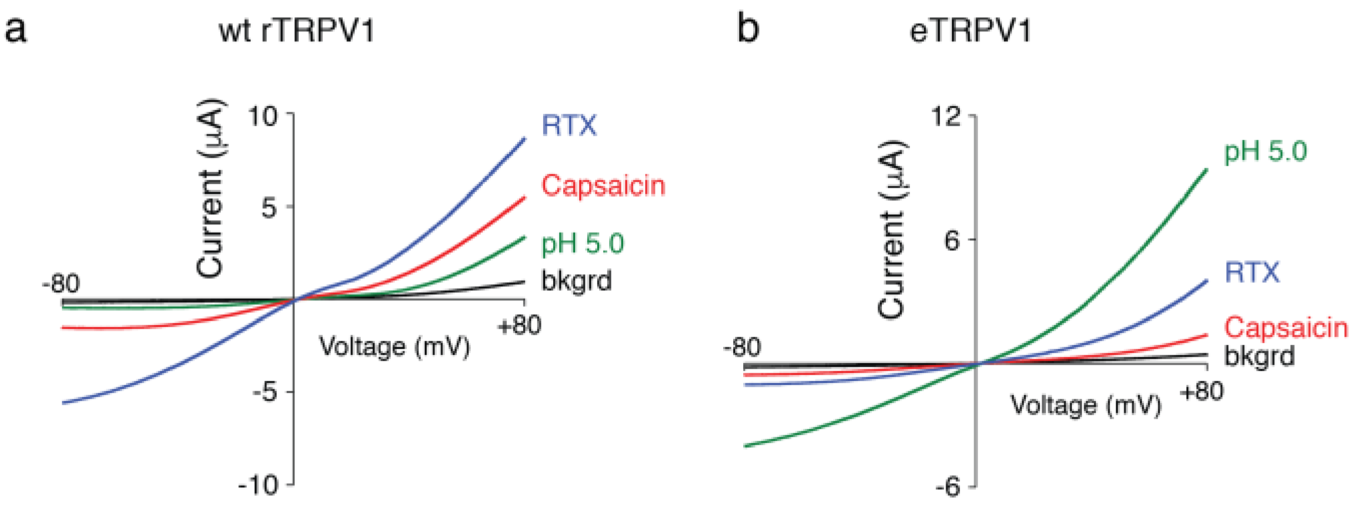

Figure S1. Related to Figure 1. Figure S1. (a and b) Current-voltage relationships of wt TRPV1 and eTRPV1 challenged with pH 5, resiniferatoxin (RTX, 10 µM), and capsaicin (Cap, 10 µM), measured using two-electrode voltage-clamp (TEVC) in *Xenopus* oocytes. Background currents (bkgrd).

Supplementary Figure S2

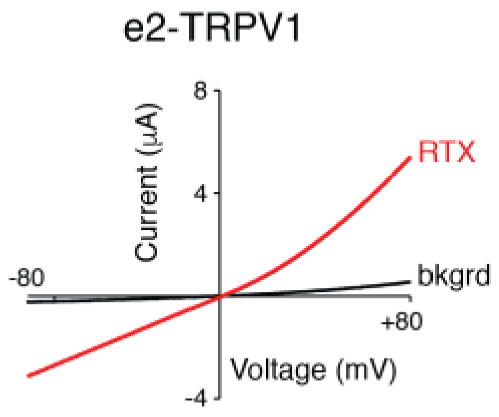

Figure S2. Related to Figure 4. Current-voltage relationships of e2-TRPV1 challenged with RTX (10 μM).
